# CUILESS2016: a clinical corpus applying compositional normalization of text mentions

**DOI:** 10.1186/s13326-017-0173-6

**Published:** 2018-01-10

**Authors:** John D. Osborne, Matthew B. Neu, Maria I. Danila, Thamar Solorio, Steven J. Bethard

**Affiliations:** 1 University of Alabama at Birmingham, 7th Ave S, Birmingham, 1720 USA; 2 Computer Science Department, University of Houston, Düsternbrooker Weg 20, Houston, 24105 USA; 3 School of Information, University of Arizona, Tucson, 85721 USA

**Keywords:** NLP, Information extraction, Concept normalization, Concept recognition, Fine grained named entity recognition

## Abstract

**Background:**

Traditionally text mention normalization corpora have normalized concepts to single ontology identifiers (“pre-coordinated concepts”). Less frequently, normalization corpora have used concepts with multiple identifiers (“post-coordinated concepts”) but the additional identifiers have been restricted to a defined set of relationships to the core concept. This approach limits the ability of the normalization process to express semantic meaning. We generated a freely available corpus using post-coordinated concepts without a defined set of relationships that we term “compositional concepts” to evaluate their use in clinical text.

**Methods:**

We annotated 5397 disorder mentions from the ShARe corpus to SNOMED CT that were previously normalized as “CUI-less” in the “SemEval-2015 Task 14” shared task because they lacked a pre-coordinated mapping. Unlike the previous normalization method, we do not restrict concept mappings to a particular set of the Unified Medical Language System (UMLS) semantic types and allow normalization to occur to multiple UMLS Concept Unique Identifiers (CUIs). We computed annotator agreement and assessed semantic coverage with this method.

**Results:**

We generated the largest clinical text normalization corpus to date with mappings to multiple identifiers and made it freely available. All but 8 of the 5397 disorder mentions were normalized using this methodology. Annotator agreement ranged from 52.4% using the strictest metric (exact matching) to 78.2% using a hierarchical agreement that measures the overlap of shared ancestral nodes.

**Conclusion:**

Our results provide evidence that compositional concepts can increase semantic coverage in clinical text. To our knowledge we provide the first freely available corpus of compositional concept annotation in clinical text.

**Electronic supplementary material:**

The online version of this article (doi:10.1186/s13326-017-0173-6) contains supplementary material, which is available to authorized users.

## Background

Post-coordinated concepts are concepts represented by combining multiple concepts from an ontology, in contrast to pre-coordinated concepts, which are explicitly predefined and represented in an ontology by a single identifier. Post-coordinated concepts have been used by medical ontological systems such as GALEN [1] and SNOMED CT [2] to elucidate a broader range of concepts than is possible with pre-coordinated systems [3, 4] using descriptive logic. This methodology relies on a restricted set of pre-defined semantic relationships to avoid or minimize semantic ambiguity. This is in contrast to Gene Ontology [5], which until the recent introduction of annotation extensions [6], assigned multiple annotations to a single protein without regard to the relationships between the assigned annotations. Not requiring formal semantic relationships for all multi-concept annotations may introduce some semantic ambiguity, but allows higher semantic coverage in situations where the source text describes a concept whose logical description cannot be captured by the set of pre-existing semantic relationships. Indeed, the ideal that an ontology of medicine can express “all and only what is medically sensible” has been termed “unobtainable” and focusing on “all” rather than “only” should take precedence [7].

In clinical interface systems utilizing SNOMED CT, complicated clinical concepts are typically created by clinicians who select from a set of inter-related atomic concepts with pre-defined relations. However the creation of a publicly available clinical text corpus with post-coordinated normalization training data has received less attention. This is likely due the difficulty and cost of creating and sharing such a corpus. Moreover, earlier work [8] comparing normalization between different SNOMED CT encoding groups that applied post-coordination to normalize text mentions in case report forms failed to find any statistically significant semantic agreement.

More recently, post-coordination has been applied in biomedical corpus construction with the creation of the NCBI Disease Corpus [9]. During corpus creation, Dŏgan first attempted to normalize disease mentions in PubMed abstracts to the MEDIC vocabulary using pre-coordinated concepts, which was successful for 91% of the disease mentions. For the remaining 9% of disease mentions, they employed a minimally restricted form of post-coordination that we term *“compositional”* normalization that allowed the use of multiple concepts without regard to specific relations or “slots”. They further categorized these *“compositional* concepts between *“aggregate”* or “composite” concepts that consisted of multiple self-contained pre-coordinated concepts in the text mention and *“composed”* concepts which collectively act to describe a single concept. The aggregate concepts in this context are simply concepts linked by logical operators (AND/OR) since no provision was made for logical operator usage in the annotation. Examples are shown in Table 1.

**Table 1:** Examples of pre-cordinated and post-coordinated concepts from the NCBI disease corpus

Type / Subtype	Identifiers	Text mention example	Concept name/s
Pre-coordinated	1	Bone dysplasia	Bone diseases, Developmental
Compositional / Aggregate (|)	2	Breast or ovarian cancer	Breast cancer|Ovarian cancer
Compositional / Composed (+)	3	Inherited neuromuscular disease	Neuromuscular disease + Genetic diseases + Inborn

Post-coordinated concepts of type (“aggregate” or “composed”) have 2 or more identifiers

In the NCBI Disease corpus, only 76 such unique compositional concepts were normalized (52 aggregate and 24 composed) and annotator agreement for these post-coordinated concepts was not reported separately.

In contrast to the open-ended nature of Dŏgan’s compositional concepts, Roberts [10] annotated post-coordinated concepts for only one predefined relation: anatomical location. Roberts’ work includes both a corpus annotated on medical consumer language and software to normalize text mentions. However, the corpus contains only 500 post-coordinated concept instances.

SemEval-2015 Task 14 [11] annotated a corpus of clinical text with post-coordinated concepts, normalizing each disorder mention to a single SNOMED CT concept, and restricting further post-coordination to 8 predefined relations: body locations, which were normalized to UMLS anatomical concepts, and 7 other small-domain concept types. We refer to this corpus as “SEMEVAL2015”. The SEMEVAL2015 section of Table 2 shows examples of each predefined relation. However, they report annotator agreement only for disorder mention normalization, not the overall normalization annotator agreement for that mention which would include associated post-coordinated concepts or slots. They were also unable to normalize 30% of the disorder mentions (such mentions are termed “CUI-less”) because annotators were unable to find a single UMLS Concept Unique Identifier (CUI) for the concept. This suggests that there are limitations in the annotation process, the ontology being normalized to (SNOMED CT) or both, which prevent the full semantic capture of clinical text. This is known as the *content completeness problem*, first coined by Elkin [12, 13] but recognized earlier by Rogers and Rector [14].

**Table 2:** CUI-less examples from SEMEVAL2015 and CUILESS2016 annotation of ShARe corpus

		Aggregate example	Composed example
SEMEVAL2015	*Text mention*	*RRW*	*Surgical defect*
	Negation	Yes	No*
	Subject	Patient*	Patient*
	Uncertainty	No*	Yes
	Course	Unmarked*	Unmarked*
	Severity	Unmarked*	Unmarked*
	Conditional	False*	False*
	Generic	False*	False*
	Body location CUI	C0225754 (Both lungs)	C1521748 (Entire mastoid)
	Disorder CUI	CUI-less	CUI-less
CUILESS2016	Disorder CUI	C0034642 (Rhales)	C0543467 (Operative surgery)
		C0035508 (Rhonchi)	C2004491 (Cicatrix)
		C0043144 (Wheezing)	

An * indicates the default value for that slot in SEMEVAL2015. Our CUILESS2016 annotators added identifiers to describe the disorder when the Disorder CUI was marked “CUI-less” in SEMEVAL2015

In the current study we evaluate the extent to which compositional annotation, not restricted to a predefined set of relations, can attenuate the content completeness problem in clinical text. To address this problem, we generate the largest corpus to date for this compositional method. To our knowledge it is the first such compositional corpus in clinical text.

## Method

### Corpus generation

We generated a novel dataset “CUILESS2016” derived from the part of ShARe corpus used for the SemEval-2015 Task 14 Shared Task [11], which we term, “SEMEVAL2015”. Only a subset of SEMEVAL2015 was utilized, consisting of those disorder mentions that were not normalized to SNOMED CT, so called “CUI-less” disorders because they lack a Unified Medical Language System (UMLS) CUI corresponding to a SNOMED CT concept. Their distribution in the SEMEVAL2015 training and development datasets is shown in Table 3. We re-annotated only the CUI-less disorder CUI; CUI-less body locations or other relations are not re-annotated, as shown in Table 2.

**Table 3:** SEMEVAL2015 CUI-less distribution by clinical document type

Data set	Document type	CUI-less count	Average CUI-less by Note
Development	Discharge summaries	1929	13.9
Training	Discharge summaries	2796	20.6
Training	Echocardiogram	331	6.1
Training	Electrocardiogram	91	1.7
Training	Radiology	250	4.6

Only discharge summaries were available for annotation in the development document set

Since test data was not readily available, only disorder mentions from the development and training portion of SEMEVAL2015 were normalized. Approximately 30% (5397) of disorder mentions fit this “CUI-less” description from a set of 298 training notes and a set of 133 development notes. The 298 training note set was itself derived from the notes used in the ShARe/CLEF eHealth 2013 Evaluation Lab Task 1 [15]. Statistics for the input SEMEVAL2015 corpus are provided in Table 4.

**Table 4:** SEMEVAL2015 and CUILESS2016 document statistics

Set	Word count	Clinical note count			
		Discharge	ECG	EKG	Radiology
Train	182K	136	54	54	54
Development	153K	133	0	0	0
Total	335K	269	54	54	54

### Annotation method

We used an open-ended compositional annotation methodology similar to that of Dŏgan [9] to normalize all 5397 “CUI-less” disorder mentions as described in the Annotation Guidelines (Additional file 1). Examples of our annotations are shown in the CUILESS2016 section of Table 2. Rules for annotation were similar to the ShARe/CLEF corpus [15] in that disorders were normalized to UMLS CUIs from SNOMED CT using the most specific CUI possible, ignoring negation and temporal modifiers, including acronyms, abbreviations and, to the fullest extent possible, mentions that are co-referent or anaphoric. There are some critical differences between the ShARe/CLEF annotation and our method that allow us to annotate these additional mentions. They are:

1. One or more identifiers were selected to annotate the text mention if (and only if) no appropriate single identifier (pre-coordinated term) is found.
2. All of SNOMED CT was available for mention normalization.
3. The annotators could use existing SEMEVAL2015 identifiers to create compositional concepts.

For example, if the mention “no bowel wall thickening” was annotated, and no CUI in SNOMED CT existed for “bowel wall thickening”, but the SEMEVAL2015 annotations include a body location CUI for “bowel wall” and the disorder was flagged as negated, then the text mention was normalized using just the CUI for “Thickened (fndg)”, since the other two concepts needed for post-coordination are already present in the SEMEVAL2015 annotations.

Unlike the work of Dŏgan [9], we made no distinction as to whether the multiple CUIs used to annotate the span were aggregate or composed concepts. Thus, all of the CUIs in our mention were space separated and could represent either aggregation (|) or concatenation (+) per the operator nomenclature of Dŏgan [9].

### Calculation of annotator agreement

Annotator agreement between the 2 annotators (MID and MN) on the development data set was computed in 2 different ways.

1. Exact Agreement - Annotators used exactly the same set of CUIs to annotate the disorder text mention. We report only proportional agreement *p*_*a*_ for this task by which we mean the fraction of *text mentions* on which the annotators agree. Thus, in Table 5 (in the Exact agreement row) we count only a single agreement for both Drug Allergy and Levofloxacin, not 2 agreements. Proportional agreement can be defined more formally as *p*_*a*_=*m*/*n* where *m* is the number of mentions where both annotators agree and *n* is the total number of mentions. This should approximate Cohen’s *κ* because agreement due to chance is expected to be extremely small. This is due to the UMLS representation of SNOMED CT having over 320K distinct CUIs and we allow an unbounded number of CUIs per mention.
2. Hierarchical Agreement - We compute hierarchical agreement between annotators using the set of annotated nodes and all their ancestors similar to the hierarchical precision and recall metric used by Verspoor [16]. It is calculated as:
  1 $$ \frac{1}{n} \sum_{i=1}^{n} (\{\uparrow A_{i}\}\cap\{\uparrow B_{i}\}) / (\{\uparrow A_{i}\}\cup \{\uparrow B_{i}\})  $$
  where {*↑A*_*i*_} indicates the set of annotated nodes and their ancestors from annotator A for mention i, {*↑B*} indicates the set of annotated nodes and their ancestors from annotator B for mention *i* and *n* is the total number of mentions annotated. In cases where an annotated CUI mapped to multiple SNOMED CT identifiers, SNOMED CT ancestors from all paths were used.

**Table 5:** CUILESS2016 annotator agreement type examples

Exact mention score	Hierarchical mention score	Text mention	Annotator 1 Concept/s	Annotator 2 Concept/s
1.0	1.0	*Allergies Levofloxacin*	Drug allergy	Drug allergy
			Levofloxacin	Levofloxacin
0.0	0.52	*Posturing*	(O/E) - posturing	Posturing behaviour
0.0	0.64	*Rightward shift*	Midline shift of brain	Midline shift of brain
			To the right	
0.0	0.22	*Redness*	Erythema	Redness

The computed hierarchical mention score was used instead of annotator judgment in determining an approximate level of agreement

### Software and data

**Fig. 1:**
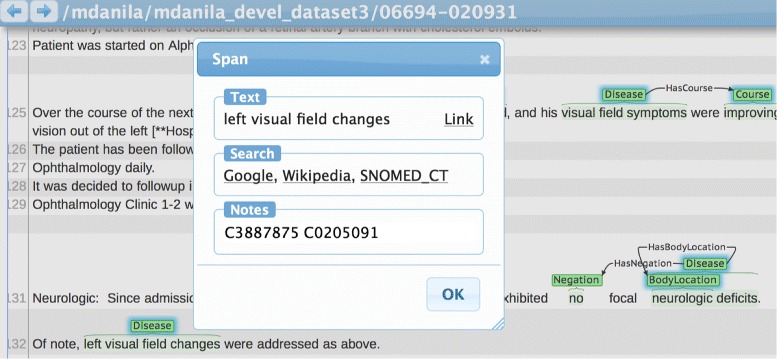
Annotation Workflow. BRAT 1.3 [17] used to normalize concepts to UMLS CUIs from SNOMED CT

Annotations were mapped using BRAT 1.3 software as shown in Fig. 1 [17]. Annotators SP, ES, MN and MID normalized the training data to the US Edition of SNOMED CT (2013_03_01) as represented in UMLS 2013AB. Development data was normalized to SNOMED CT (2016_03_01) in UMLS 2016AA by annotators MID and MN. Disorder CUIs found in the training data that were not present in SNOMED CT 2016_03_01 due to vocabulary changes or errors in the original annotation were normalized to SNOMED CT (2016_09_01) by MID and JDO.

## Results

As shown in Table 6 we found the majority of disorder mentions had only a single identifier, which reflects the expanded range of available concepts and our guidance to use pre-coordinated concepts preferentially as outlined in our annotation guidelines. However Table 6 under-represents the true disorder multi-identifier count since disorder CUIs can be post-coordinated with SEMEVAL2015 annotations that represent disorder attributes. Thus “no bowel wall thickening” would be counted as “Single” in Table 6 since only the identifier for “Thickened (fndg)” was directly annotated; the anatomical CUI and negative polarity were already present in the linked SEMEVAL2015 attribute annotations.

**Table 6:** Disorder multiple identifier distribution by data set

Disorder CUI type	Development count	Development proportion	Training count	Training proportion
CUI-less	1	0.05	7	0.20
Single	1687	87.46	2823	81.40
Double	221	11.46	562	16.21
Triple	18	0.93	73	2.11
Quadruple	2	0.10	3	0.09
Total	1929	100	3468	100

Differences in disorder mention distribution between the development and training data set are likely due to note composition (see Table 3), a larger (4) set of annotators in the training data and a lack of a consensus process for the training data since each training document is annotated only by a single annotator

Table 7 shows the overall distribution of disorder-related identifiers both when attributes (non-disorder identifiers assigned in SEMEVAL2015) are either included or excluded from consideration. Thus in the **Disorder + Attributes** column the text mention “no bowel wall thickening” was scored as having 3 identifiers, one for the disorder, one for the anatomical location and one for negation. Only when including these attributes are the majority of the concepts in CUILESS2016 post-coordinated.

**Table 7:** Overall disorder and attribute multiple identifier distribution

Identifier type	Disorder		Disorder + Attributes	
	Count	Proportion	Count	Proportion
CUI-less	8	0.1%	3	0.06%
Single	4502	83.54%	966	17.90%
Double	783	14.53%	2505	46.41%
Triple	91	1.7%	1608	29.79%
Quadruple	5	0.1%	263	4.87%
Pentuple	0	0.0%	50	0.93%
Hextuple	0	0.0%	20	0.04%
Total	5397	100%	5397	100%

The **Disorder** column shows the count and proportion of disorders annotated with one or more concepts excluding attributes. The **Disorder + Attributes** column includes identifiers from attributes in the count to capture post-coordination with other identifiers

Annotator agreement on the development set is shown in Table 8.

**Table 8:** Development dataset annotator agreement

Agreement type	Agreement count	Proportionate agreement
Exact	1011	52.4
Hierarchical	NA	78.2
Total mentions	1929	

There is no count for hierarchical agreement since each mention is assigned a value based on Eq. (1), whereas exact agreement assign every mention as a match (1.0) or not (0.0)

## Discussion

We have normalized all but 8 of the 5397 original “CUI-less” concepts in our corpus indicating that a compositional normalization methodology can alleviate the “content completeness problem” and increase semantic coverage in clinical text. All examples where our approach failed to normalize concepts are shown in Table 9. These examples fall into 3 general classes, those where the entity is not really a disease (named entity recognition failure), those where the text is ambiguous, and those where the annotators were unable to find a suitable composition in SNOMED CT. Only the last of these classes represents a concept that was truly not normalizable under our methodology. The 3 cases that fall into this class represent a tiny fraction (0.06%) of the original 5397 mentions. Leveraging the existing SEMEVAL2015 annotation (which specified 8 different semantic modifiers of disorders) and allowing our annotators to normalize using a general semantic “association” (without specifying the exact relationship) allowed us to dramatically increase semantic coverage. Our corpus should be of interest to developers of clinical text normalization software interested in annotating a wider range of disorder annotations. We make our corpus freely available.

**Table 9:** Compositional CUI normalization error analysis

Mention	Error Class
Allergies, Calcium	Named entity recognition failure
Atrial sensed	Named entity recognition failure
Left ventricular inflow pattern	Named entity recognition failure
RCIA	Ambiguous text
RC one Aneurysm	Ambiguous text
Echogenic kidney	No composition found
Making grammatical errors	No composition found
Tortous aorta	No composition found

All 8 mentions where annotators were unable to annotate the disease using the compositional approach

While our methodology is similar to that used by Dŏgan [9] for PubMed abstracts, we provide an order of magnitude more compositional normalization data. With the exception of some common abbreviations, the majority of compositional clinical concepts we created are composed concepts, not aggregate concepts. This is in sharp contrast to Dŏgan [9] where the majority of mentions (114) from PubMed abstracts are aggregates of discrete concepts and only 34 mentions (24 unique) require logical description. Moreover, a substantial proportion (at least 16%) of the CUI-less clinical concepts required compositional normalization to specify the disorder mention. This is a higher proportion than is seen previously in PubMed abstracts [9] and consistent with the greater variability of clinical text.

### Exact annotator agreement

There is a clear need for multi-identifier annotation in the clinical arena, where multiple identifiers are semantically critical for diseases such as cancer [18] and peripheral arterial disease [19]. However, evaluating the annotator agreement of post-coordinated concepts is difficult because of a lack of a common annotation standard. Previous studies reported proportionate agreement on exact matches [8, 15, 20], but the definition of an “exact match” can vary.

For example Andrews [8], took research questions from case report forms and provided them to 3 different coding companies and instructed them to extract (normalize) core SNOMED CT concepts, using either pre-coordinated or post-coordinated expressions. Normalization was measured using proportionate agreement only at the “core concept” level, which ignored disagreements resulting from additional identifiers from modifiers. Even with this restriction, agreement between all 3 coding companies was calculated to be only 33%, with 44% agreement between the two most similar annotation sets. Using Krippendorff’s *α* as their statistic they concluded there was no significant semantic agreement in normalization. In contrast, our proportionate exact agreement (our worst performing metric) was 10% higher than their best inter-annotator agreement although we were more stringent in including disagreement to extend to non-core concepts. This may be due to their data set which was focused on rare diseases in case report forms (rather than clinical text), differences in the tool selection and/or annotator medical knowledge.

An alternative measure of annotator normalization agreement (accuracy) was used in the original annotation of this corpus [15] instead of Cohen’s *κ* and Krippendorf’s *α*. Annotator normalization agreement was calculated between annotators and was not separated from the underlying mention span boundary detection. A relaxed accuracy calculation where correctness was defined as any overlapping span where the disorder CUIs matched yielded an accuracy of 0.776, a “strict” agreement score based on exact span matching yielded a much higher agreement of 0.846. However this high accuracy applies to single CUI disorder agreement. No annotator agreement was reported including disagreements with CUIs from the body location attribute or other included identifiers. While that reported “exact” agreement is higher than ours, we expected our agreement to be substantially lower since our annotation was for “CUI-less” disorders that they did not annotate. The original annotation deliberately excluded use of the UMLS semantic group finding for these disorders and reported that “this semantic group was found to be a noisy, catch-all category, and attempts to consistently annotate against it did not succeed in our preliminary studies.”

### Non-exact annotator agreement

Our exact agreement calculation cannot determine if a pre-coordinated concept and a post-coordinated concept are logically equivalent. Additionally, exact agreement cannot capture the difference between concepts with completely different meanings and hyponyms/hypernyms that have similar meanings. Our hierarchical agreement measure can account for this distinction. Hierarchical agreement penalizes distant errors and those at the higher levels of the hierarchy more severely than finer misclassifications, similar to hierarchical precision [16]. Unfortunately, the performance of hierarchical agreement is dependent on the structure of the ontology used. It is sensitive to the level of branching and assumes a consistent correlation between branch length and semantic distance. Thus even semantically similar concepts such as the posturing example seen in Table 5 may not score well, a consideration given the semantic duplication in SNOMED CT [21, 22]. We thus asked our annotators to consider the sets of concepts in each disagreement, and judge whether they were semantically equivalent, using their knowledge as medical professionals, rather than the exact structure of the ontology. The two annotators reached consensus easily on this task; there was only one case where they could not reach consensus, and for this, a neurologist was consulted to resolve the dispute. This process yielded a “semantic agreement” level of 71.6%, 19% increase over our exact agreement and is consistent with Casper [20] who reported 53% exact agreement and 75% semantic agreement.

### Compositional annotation rules

One unresolved consideration with compositional annotations is which rules or conditions should govern annotation construction. In a previous study [8], the 3 coding companies mapping to SNOMED CT presumably (not specified in paper) used the extremely structured and elaborate SNOMED CT specific post-coordination specification to compose any post-coordinated diseases they annotated. However Pradhan [15] took a more general (but domain specific) approach specifying only 9 permissible disorder modifiers. All of these disorder specific domains (with the exception of body location) had a small (single digit) range of acceptable values. While core disorder concepts annotated in these publications should be comparable, associated concepts should be expected to be quite different. The more general annotation approach taken by Dŏgan [9] and this work allowed for any concept within the target ontology or ontologies. This allows for more flexibility at the expense of interpretation. For example, a body location CUI could refer to the site of disease finding, an affected organ, or a procedure site related to the illness. It is an open-ended question whether it is better to define the set of rules and allowable domains for post-coordination for each domain or to allow unrestricted composition. An enumerated set of possible relationships make closed world logic operations possible, but enumerating a complete and useful set of distinct semantic relationships that can be described in natural language text may not be feasible [7].

### Practical applications

A practical application of our work is increasing semantic representation in clinical text. The approximately 70% coverage of named entities in SemEval-2015 Task 14 is too low for many practical purposes. Additionally, while SEMEVAL2015 corpus has the most exhaustive set of relations or slots for diseases to date, it still does not include important clinical relationships useful for practical applications of NLP. For example, metastasis, infection, surgical procedures or other SNOMED CT specified relations are relevant for practical clinical use. Additionally, by creating a corpus that includes clinical compositional annotation, this corpus opens the door to such annotation by machines that could potentially reduce the clinical coding burden.

### Limitations

We have shown that annotating text from discharge summaries with compositional concepts from SNOMED CT is possible with high levels of annotator agreement. While this approach improves semantic coverage and is not bound to specific semantic relationship types, it does introduce a measure of semantic ambiguity since the relationship between the concepts is unclear. Thus, our annotations are more useful for information extraction than for logical reasoning, especially since we do not annotate logical operators (AND/OR) which would be useful in distinguishing aggregate from composite concepts. Future work should be able to make this distinction and also determine if our results are achievable for other medical text types (e.g., pathology reports) and other medical ontologies (e.g., the consumer health vocabulary).

We have shown high annotator agreement for annotating a single text mention with the identifiers of multiple ontological concepts, though we expect this agreement is lower than agreement on single identifier mentions. Unfortunately, we are unable to directly calculate single-identifier agreement because, under our annotation scheme, a mention which has been annotated with a single identifier may represent either (1) a true single-identifier disease/disorder where the identifier completely captures the meaning, or (2) a disease/disorder where a single identifier captures only part of the meaning but the remaining meaning is captured by linked attributes (e.g., the body location already identified by the SemEval-2015 Task 14 annotations).

## Conclusions

In conclusion, we extended the SemEval-2015 Task 14 annotations of the ShARe disorder corpus to cover “CUI-less” concepts and showed that the compositional annotation approach first used by Dŏgan [9] on PubMed text can function in clinical text to assign semantic identifiers to named entities and reduce the “content completeness problem” [12, 13]. We believe our larger, freely available corpus is an important resource for the annotation of “CUI-less” concepts and that information extraction utilizing compositional normalization can lead to a more complete understanding of clinical text by complementing annotation approaches using pre-defined relations or slots such as the original ShareClef annotation. While annotation of complex clinical concepts using multiple identifiers has been routinely done by humans in a clinical or research setting, this corpus should aid the development of compositional normalization by machines to supplement manual coding practises.

## Additional file

Additional file 1 Annotation Guidelines for Annotating CUI-less Concepts in BRAT. (PDF 1050 kb)
