# Supplementary material for: CUILESS2016: a clinical corpus applying compositional normalization of text mentions
Source: J Biomed Semantics. 2018 Jan 10;9:2. doi: 10.1186/s13326-017-0173-6 (PMC5761157; doi:10.1186/s13326-017-0173-6)
Supplement: Additional file 1 — Annotation Guidelines for Annotating CUI-less Concepts in BRAT. (PDF 1050 kb) [file 13326_2017_173_MOESM1_ESM.pdf]

Annotation Guidelines for Annotating CUI-less Concepts in BRAT

John David Osborne

Mar 8, 2015

Contents

1 Overview **1**

2 Annotating Text **2**
2.1 Overview of CUI Assignment 2
2.2 CUI Selection Guidelines 2
2.3 Examples with Text 3
2.4 Short Examples 3
2.5 Example Walkthrough 4
2.5.1 "Unable to walk" 4

3 Navigation **7**
3.1 Setting up and using the SNOMED CT Browser 7
3.2 Setting up and using BRAT 9

1 Overview

The goal of this annotation is map text from clinical documents (Discharge Summaries, ECG Reports and Echocardiograms) to SNOMED CT concepts in UMLS. Concepts in UMLS are identified with a unique identifier called a "CUI" that starts with the letter C, for example "C0043395" is the CUI for yellow fever. In some cases, we will map a single highlighted piece of clinical to text to more than 1 CUI. Everything you need to use to do the annotation can be done in a web browser, but you will **need to use Google Chrome**. You will need to have at least 2 tabs in Google Chrome open, one to look at the clinical text through an annotation tool called BRAT and another to look up concepts using the the NLM SNOMED CT browser. You might need a 3rd window to google words or abbreviations for which you don't know the meaning.

This document has 2 main sections, the first section ("Annotating Text") describes the annotation process conceptually and the last section ("Navigation") provides step by set instructions on how to log in and use BRAT and the SNOMED CT Browser.

2 Annotating Text

2.1 Overview of CUI Assignment

The annotation goal is to assign one or more CUIs to the highlighted disease/disorder text by adding the CUI to the "Annotator Notes" section of the highlighted concept. Added CUIs should be separated by spaces and should contain at least CUI with a semantic type from the UMLS disorders group since that is the criteria by which they were originally identified. Examples of the Disorder Group semantic types are listed below.

1. T020|Acquired Abnormality
2. T190|Anatomical Abnormality
3. T049|Cell or Molecular Dysfunction
4. T019|Congenital Abnormality
5. T047|Disease or Syndrome
6. T050|Experimental Model of Disease
7. T033|Finding
8. T037|Injury or Poisoning
9. T048|Mental or Behavioral Dysfunction
10. T191|Neoplastic Process
11. T046|Pathologic Function
12. T184|Sign or Symptom

You can tell the semantic type of the CUI you are select is by looking at the SNOMED-CT browser, under "UMLS Information". You should see text like: "Semantic Types: Congenital Abnormality [T019]" The goal is to **completely represent** the highlighted text to the **highest degree of specificity possible** using a single vocabulary - SNOMED CT. Thus other semantic types such as (ex qualifiers) may be needed as well.

2.2 CUI Selection Guidelines

1. Pick as few CUIs as possible to represent the text
2. Match the meaning of the text as closely as possible. If more CUIs need to be added to match the meaning completely, add them. For example if the text contains body positioning concepts (left, right, inferior, superior) find concepts or qualifiers to include those.

3. When picking CUI/s to represent the text, preferentially select CUIs such as disease, pathologic function, congenital abnormality before using a more generic type like “finding”.

4. The CUIs selected should try to include all over the pre-existing disorder attributes such as body location, severity. For example, select a CUI for “breast cancer” not just cancer even if the body location “breast” is already highlighted. However if there is no CUI for “breast cancer” and the body part is already highlighted there is no need to add an additional CUI for breast.

5. Try to avoid selecting CUIs where the concept name includes "dysfunction", "abnormal" if a more specific concept can be found.

6. If no set of appropriate CUIs can be found for the concept, fill in CUI-less in the annotator notes. This should be rare.

7. If something is not a disease or medical issue then fill in NO-DISEASE instead of a CUI. This should be extremely rare.

8. If you have further questions email ozborn@uab.edu

2.3 Examples with Text

The text highlighted in red corresponds to the disease mention.

1. EMTs were called and found the patient confused and lethargic, with dried blood in his mouth. Dry (qualifier value) (C0205222) and Blood clot (morphologic abnormality) (C0302148).

2. The left atrium is elongated. Extended (qualifier value) (C0231449) and Length (attribute) (C1444754).

3. Left ventricular wall thickness, cavity size, and systolic function are normal (LVEF>55%). Due to suboptimal technical quality, a focal wall motion abnormality cannot be fully excluded. Fill in Cardiac wall motion (ortf) (C0232167) and Focal (qualifier value) (C0205234). Do not specify left ventricular wall motion abnormality if left ventricular is not specified, even if 99% of such wall motions refer to the left ventricle

4. POD three was significant for dropping oxygen saturation with increased pulmonary toilet. Use “Finding of oxygen saturation” (C3163851).

2.4 Short Examples

1. depressed LVEF. Use “Left ventricular ejection fraction (lbtr)/C0428772” and “Decrease (qnco)/C0547047”. “Left ventricular ejection fraction (lbtr)/C0428772 does not cover the text “depressed”.

2. elevated liver function tests. Use “Liver Function Tests(lbpr)" and “High(qlco)” not “Liver function tests abnormal finding(fndg)” since the latter does not specify the problem and is too general.

3. mucosal thickening. Use “Thickened(fndg)” and “Structure of mucous membrane of ethmoid sinus(bpoc)” rather than “Mucous membrane swelling” unless swelling is explicit and unambiguous from the context.

2.5 Example Walkthrough

2.5.1 “Unable to walk”

Ok, you’re logged in and your first document is in front of you. Here’s how you annotate.

1. 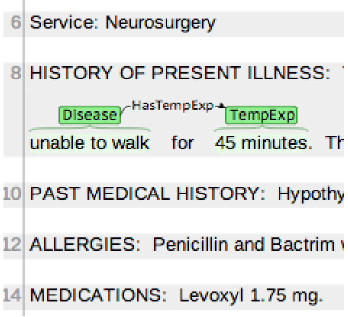

   Find the next disease concept, in this case it is the first one and the disease (we use the term loosely) is “unable to walk”. The “Disease” is highlighted in green. You’ll also notice it has a Temporal Relationship with another piece of text “45 minutes”. There are 10 different types of relationships, but the only thing you need to focus on is the Disease text. This is the text that was not mapped in the original annotation of these documents.

2. 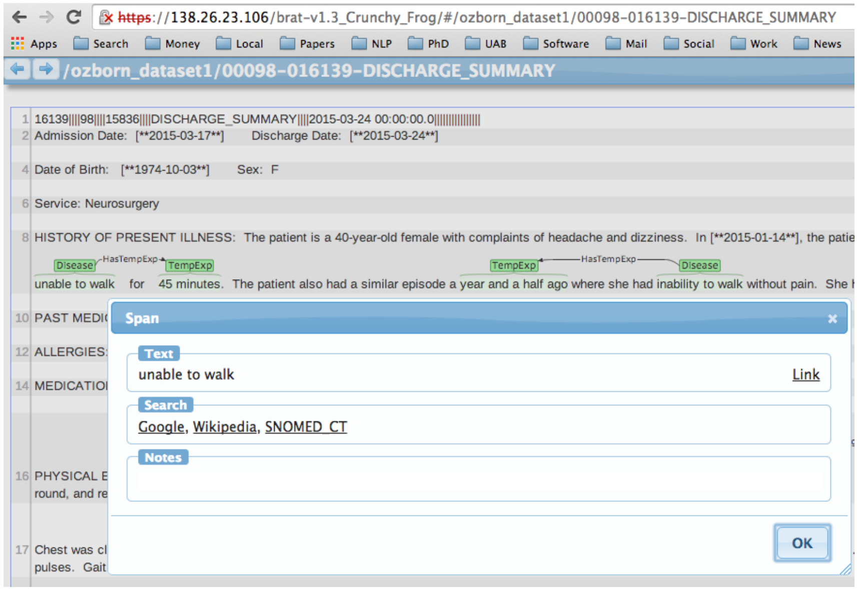

Click on the text "unable to walk". A box should popup as shown above containing the text as well as links for searching to Google, Wikipedia and the SNOMED CT Browser.

3. 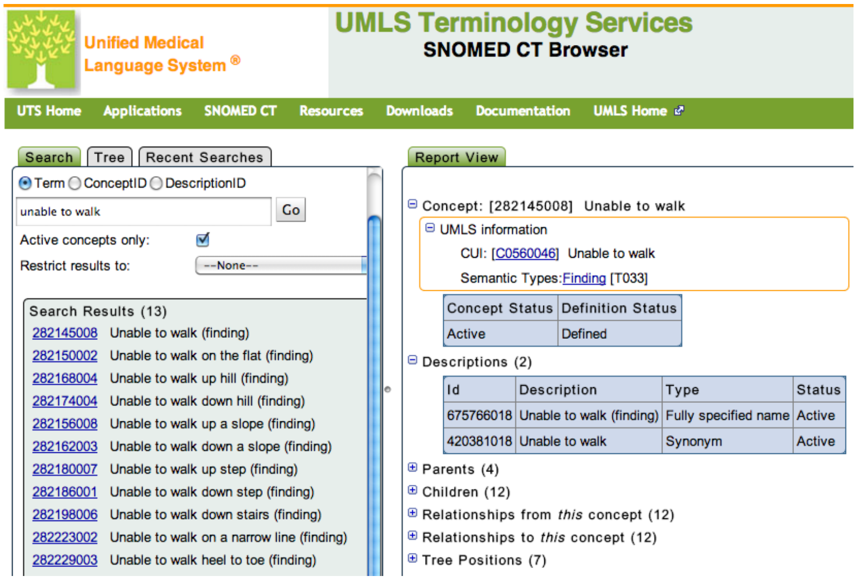

In the pop-up box, click on "SNOMED CT" (after Google and Wikipedia) to do a quick search in your browser. If you are logged in to UMLS (as described earlier), it should do a search for you and give results like above. This should be an easy search, the concept description perfectly fits the text "unable to walk".

4. 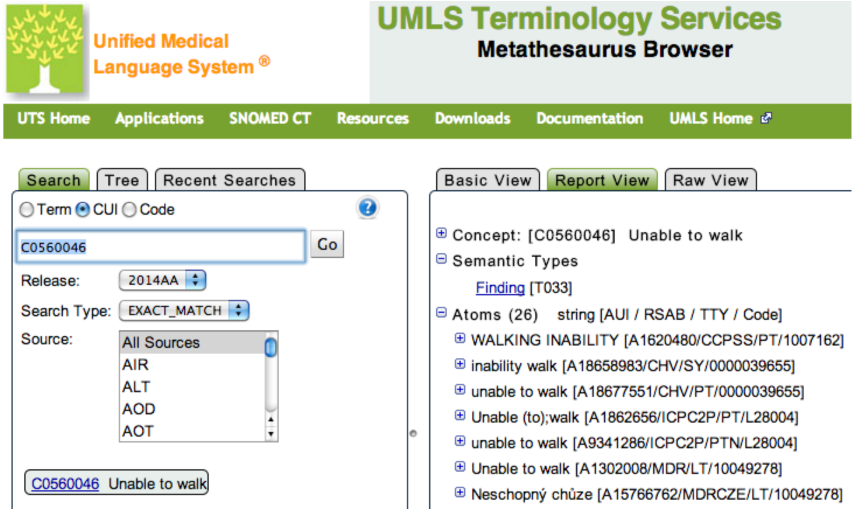

You can click on the discovered CUI (C0560046) to see multiple descriptions of that concept for different vocabularies in UMLS.

5. 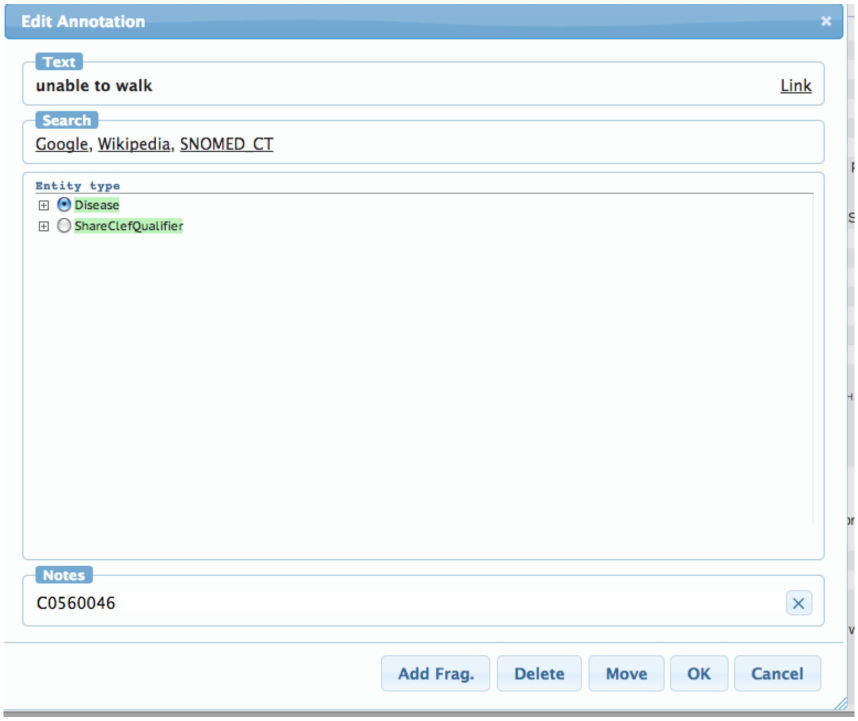

Copy the CUI from the previous window and paste it into the Notes box.

Then press "OK". Congratulations, you just annotated your first concept! If you needed additional CUIs for this concept you would have just added them in the same Annotator Note box, separated by a space.

3 Navigation

3.1 Setting up and using the SNOMED CT Browser

1. Open Google Chrome
2. Go to `https://uts.nlm.nih.gov/home.html`

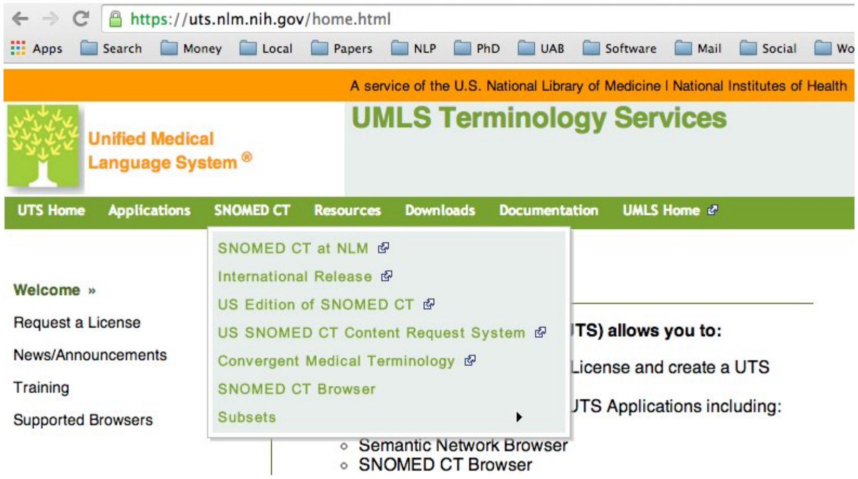

3. Click on SNOMED CT between Applications and Resources. Scroll down the pop up list and select SNOMED CT Browser.

4. 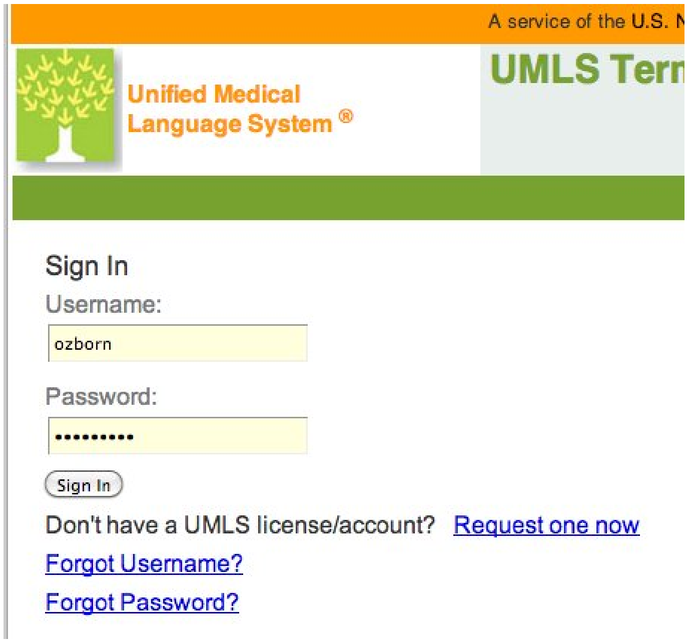

Log in to UMLS. If you have a UMLS username and password use that, otherwise you can use mine. Username:[redacted] Password:[redacted]

5. 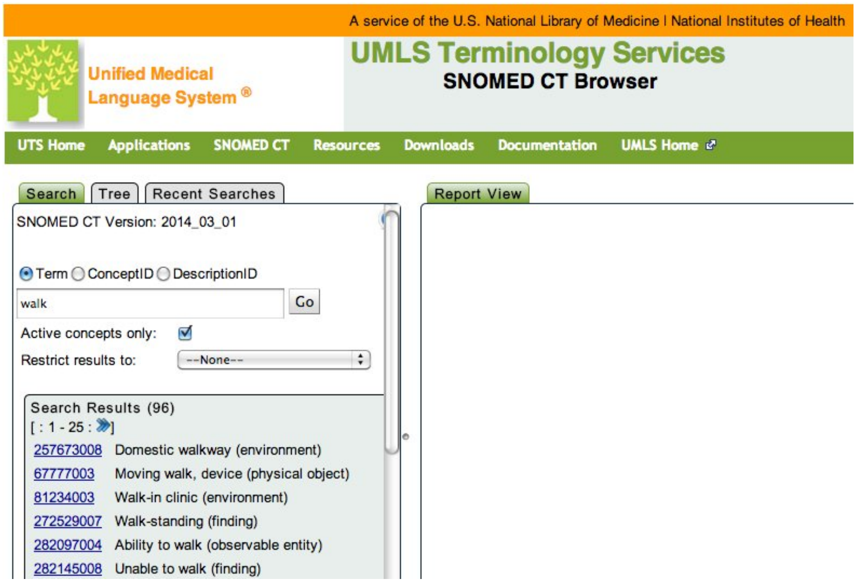

You will be using this page to type in words (such as walk shown above) into the search box in order to retrieve CUIs for annotation. Alternatively you will be able to click in the BRAT tab on SNOMED CT (shown in the first examp.e) and it will automaticaly enter the text as a search term.

3.2 Setting up and using BRAT

1. Open Google Chrome
2. Go to `https://138.26.23.106/brat-v1.3_Crunchy_Frog/#/`

3. 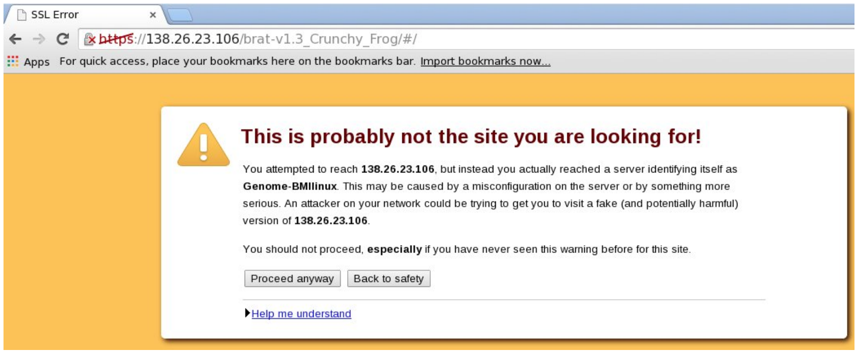

You should see an error message (shown above) indicating the webiste has no certficiate. Ignore it and click proceed. If you can't connect at all, it is possible that you are connecting from a non-UAB computer, that is one with an IP address not starting with 138.26 or 164.111. If this is the case send me email so we can add your range of IP address so you can work from home, Starbucks, etc...

4. 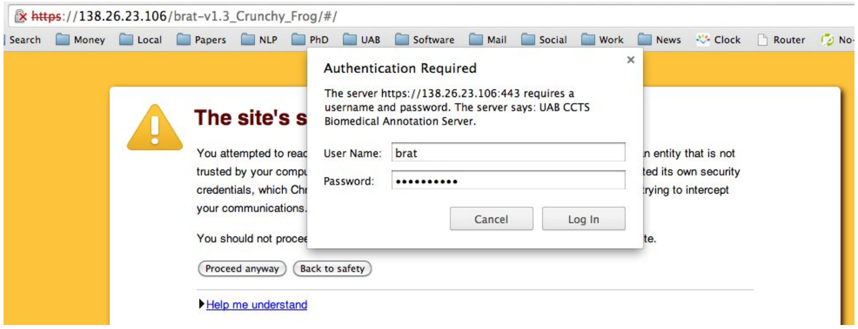

Log in to the Apache Web Server using username "brat" and password [redacted]. This username/password combination is the same for everybody.

5. 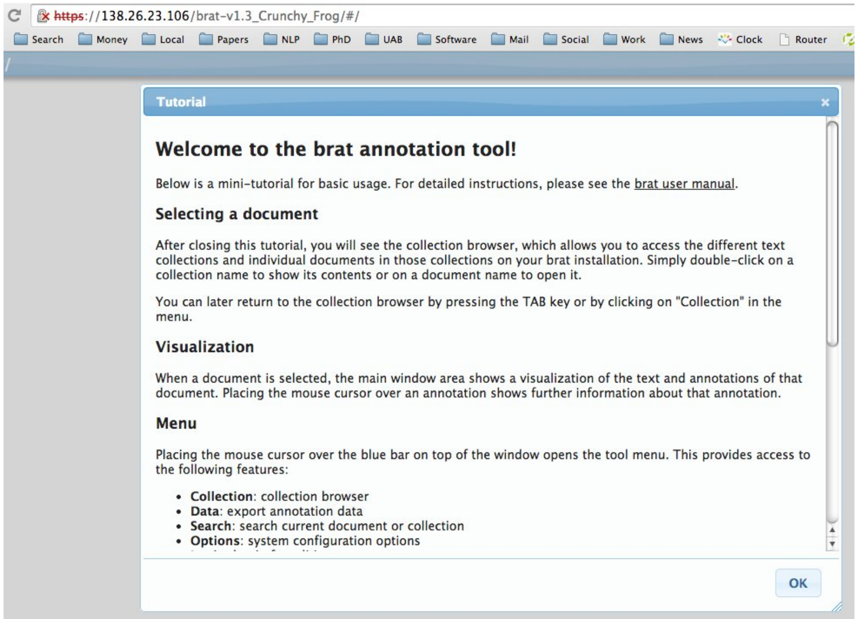

You should now see the Welcome Page shown above, which provides some information on how to use BRAT - the annotation tool we are using. You can now click OK in the bottom right hand corner.

6. 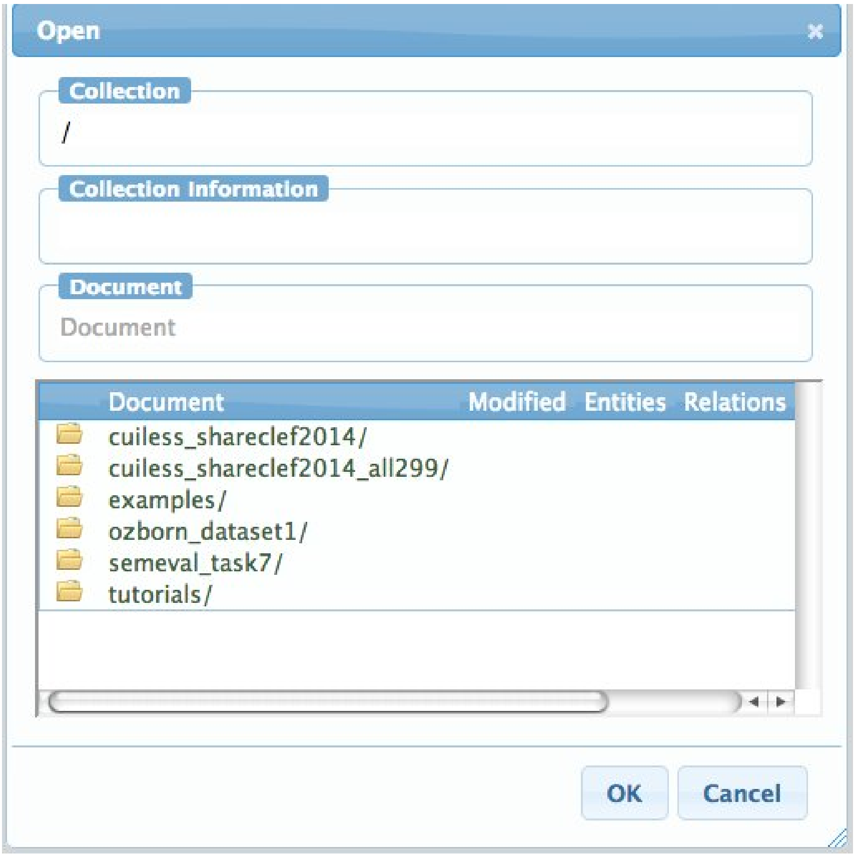

The “Welcome to the BRAT annotation tool" window should have disappeared and when it does it should reveal the collection browser consisting of a list of different datasets. Double click to open the appropriate dataset starting with your username, something like username_datasetN. The name of the dataset you need to work on should have been sent to you by email.

7. 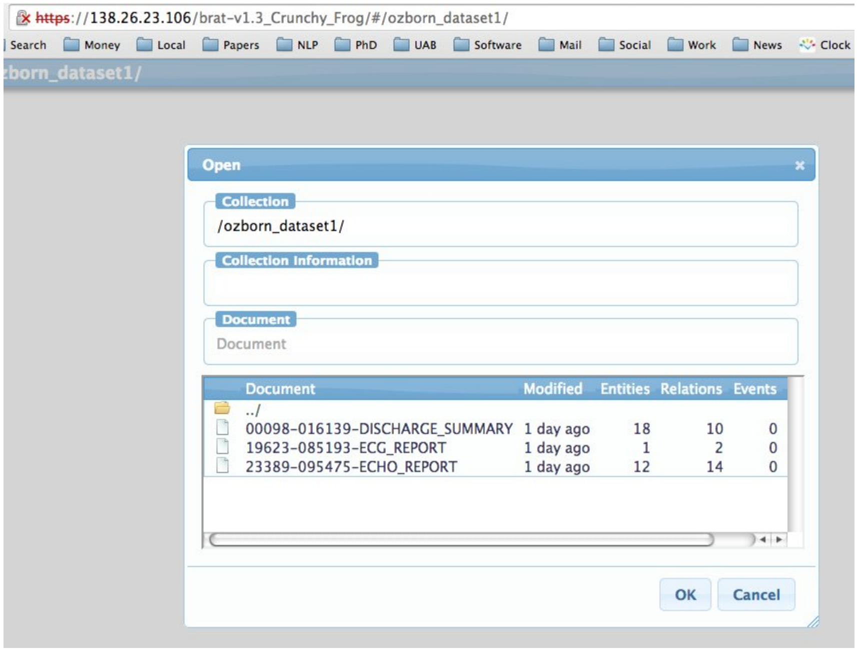

Select the 1st document in the dataset.

8. 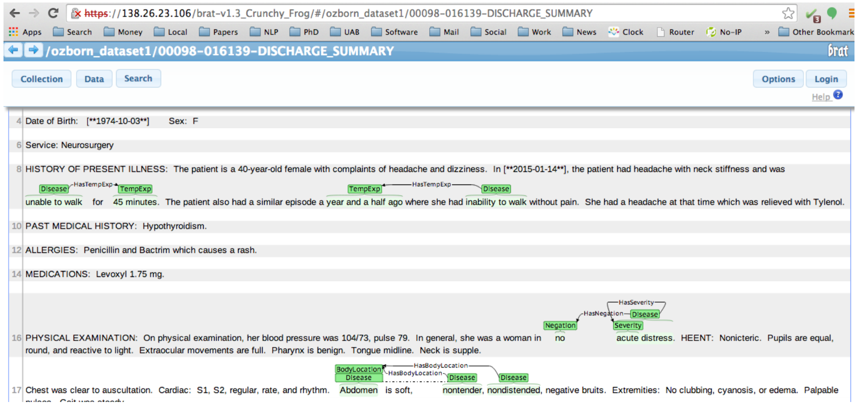

Move to the blue band where it says BRAT on the upper right hand corner. A pull down menu should appear with a login sign (but you may have to wait a few seconds).

9. 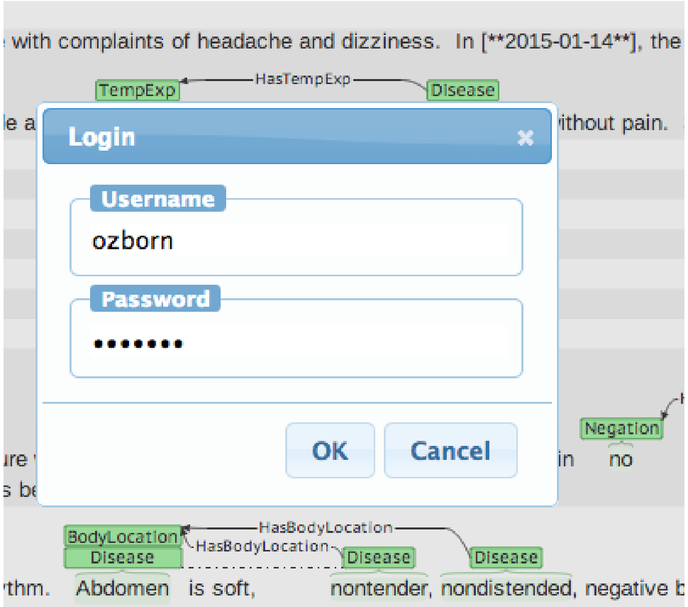

Log in using the username and password you have been provided with by email.
